# Suppression of a Field Population of *Aedes aegypti* in Brazil by Sustained Release of Transgenic Male Mosquitoes

**DOI:** 10.1371/journal.pntd.0003864

**Published:** 2015-07-02

**Authors:** Danilo O. Carvalho, Andrew R. McKemey, Luiza Garziera, Renaud Lacroix, Christl A. Donnelly, Luke Alphey, Aldo Malavasi, Margareth L. Capurro

**Affiliations:** 1 Oxitec Ltd, Abingdon, Oxfordshire, United Kingdom; 2 Departamento de Parasitologia, Instituto de Ciências Biomédicas, Universidade de São Paulo, São Paulo, Brasil; 3 Moscamed Brasil, Juazeiro, Bahia, Brasil; 4 Medical Research Council Centre for Outbreak Analysis and Modelling, Department of Infectious Disease Epidemiology, Faculty of Medicine, Imperial College London, St Mary's Campus, London, United Kingdom; 5 Department of Zoology, University of Oxford, Oxford, United Kindgom; 6 The Pirbright Institute, Pirbright, Woking, Surrey, United Kingdom; 7 Instituto Nacional de Ciência e Tecnologia em Entomologia Molecular (INCT-EM), Rio de Janeiro, Brasil; Colorado State University, UNITED STATES

## Abstract

The increasing burden of dengue, and the relative failure of traditional vector control programs highlight the need to develop new control methods. SIT using self-limiting genetic technology is one such promising method. A self-limiting strain of *Aedes aegypti*, OX513A, has already reached the stage of field evaluation. Sustained releases of OX513A *Ae*. *aegypti* males led to 80% suppression of a target wild *Ae*. *aegypti* population in the Cayman Islands in 2010. Here we describe sustained series of field releases of OX513A *Ae*. *aegypti* males in a suburb of Juazeiro, Bahia, Brazil. This study spanned over a year and reduced the local *Ae*. *aegypti* population by 95% (95% CI: 92.2%-97.5%) based on adult trap data and 81% (95% CI: 74.9-85.2%) based on ovitrap indices compared to the adjacent no-release control area. The mating competitiveness of the released males (0.031; 95% CI: 0.025-0.036) was similar to that estimated in the Cayman trials (0.059; 95% CI: 0.011 – 0.210), indicating that environmental and target-strain differences had little impact on the mating success of the OX513A males. We conclude that sustained release of OX513A males may be an effective and widely useful method for suppression of the key dengue vector *Ae*. *aegypti*. The observed level of suppression would likely be sufficient to prevent dengue epidemics in the locality tested and other areas with similar or lower transmission.

## Author Summary

Dengue is a major mosquito-borne disease, increasing in prevalence and severity; there are no specific drugs or licensed vaccine. It is primarily transmitted by one mosquito species, *Aedes aegypti*. We released transgenic ‘sterile’ male mosquitoes in Itaberaba, a suburb of Juazeiro, a Brazilian city. Sustained release of these males, whose offspring typically die before adulthood as a consequence of the transgenic modification, strongly suppressed the target wild population—by 80–95% according to different measures. These data are consistent with previous releases in the Cayman Islands, suggesting that differences between the two locations, including the environment or wild mosquito strain, made little difference. Mathematical models suggest that this degree of suppression would be highly effective in preventing epidemic dengue.

## Introduction

Dengue is second only to malaria as most important mosquito-borne disease. Unlike malaria and other major infectious diseases, dengue is increasing in incidence and severity, currently inflicting 50–390 million or more cases per year worldwide [1, 2]. Dengue is widespread in tropical and sub-tropical areas and is primarily associated with its principal vector *Aedes aegypti* (L.).

Dengue was reintroduced in Brazil in 1981 (Boa Vista, State of Roraima), after being almost entirely absent for at least 20 years following DDT-based vector control. Brazil now has serotypes 1–3 circulating throughout the country; in addition serotype 4 was recently detected in several states [3]. In an analysis of dengue in the Americas in 2000–2007, Brazil was found to have the highest number of cases and economic burden [4]; more recently [1] estimated 16 million total infections annually. While this in part reflects the size of the Brazilian population, Wilder-Smith et al. [5] concluded that the dengue burden is at least as high as the burden of other major infectious diseases that afflict the Brazilian population, including malaria. A cross-sectional seroepidemiologic survey conducted in Recife, state of Pernambuco, Brazil, in 2006 found overall dengue virus IgG prevalence to be 80% indicating that the large majority of inhabitants have been infected at least once [6]; these authors estimated that 5.2% of susceptible individuals become infected each year by each serotype and that this had increased sharply over the previous 20 years.

There are no specific drugs or licensed vaccine for dengue, so efforts to reduce transmission depend entirely on vector control [2]. However, even the most highly-resourced and well-implemented programmes, such as in Singapore, have not been able to prevent epidemic dengue using current methods [7–9]. Furthermore, existing control tools are threatened by actual or potential spread of resistance in the vector population. Therefore there is an urgent need to develop new methods. The use of transgenic vectors may provide a set of new methods for reducing the density or vectorial capacity of vector populations [10]. Here we describe a field evaluation of one prominent transgenic-vector strategy, the use of male mosquitoes carrying a lethal or autocidal transgene in a sterile-male-release system.

The Sterile Insect Technique (SIT) is a genetic control system based on the release of large numbers of radiation-sterilised insects. These mate with wild insects of the same species and thereby reduce the reproductive potential of the wild pest population, as they produce no or fewer viable offspring due to the radiation-induced presence of lethal mutations in their gametes [11, 12]. Though successfully used against several agricultural pests, trials against mosquitoes have met with less success [13, 14]. This is in part due to the somatic damage, and associated performance reduction in the sterile insects, which inevitably accompanies radiation-sterilisation. Interestingly, one successful example of SIT in mosquitoes used a chemosterilant in place of radiation [15]. Modern genetics can potentially overcome this problem, for example by using an engineered self-limiting gene, that is both repressible by an antidote provided in a managed rearing facility and when expressed in the absence of the repressor any insect carrying the gene results in mortality before the insect reaches functional adulthood, which may be used in place of radiation [16]. Operationally, the system would look very similar to SIT, and would share the clean, species-specific characteristics, and similarly benefit from the female-seeking ability of the released males. However the insects would not be irradiated, rather they would be homozygous for a transgene which, when transmitted to an embryo via the sperm, would lead to death of the zygote at some stage in development [17, 18]. As well as avoiding the need for radiation, by adjusting the time of death one can improve efficiency against target populations with significant density-dependence [19, 20]. Simulation modelling suggests that such a method would potentially be effective and economical against *Ae*. *aegypti* [19, 21].

After extensive laboratory development and testing, field testing of engineered insects has begun, with encouraging results. In particular, in the Cayman Islands a self-limiting strain of *Ae*. *aegypti*, OX513A, was shown to be able to compete successfully for wild mates, furthermore sustained release of OX513A males suppressed a wild population of *Ae*. *aegypti* [10, 22]. We tested whether this same strain and strategy could also be effective in Brazil. Within the overall project objective of evaluating OX513A technology in Brazil, we had three core technical activities. These were (i) to transfer the technology to Brazil, including adaptation and optimisation for local conditions; (ii) to assess the field performance in terms of mating competitiveness of OX513A males in Brazil; and (iii) to test the ability of OX513A males to suppress a wild *Ae*. *aegypti* population in this environment. The fourth core activity, which will be described in detail elsewhere, related to community engagement and regulatory activities.

## Methods

### Study area

The study was conducted in the Itaberaba suburb of the city of Juazeiro, Bahia in the semi-arid North East of Brazil (latitude—9.450, longitude—40.481), both treated and control sites were in the same suburb and, consequently, had similar characteristics. The site consisted predominately of housing of relatively low social economic status and was identified by local public health officials as having high dengue incidence. A dependence on stored water (due to irregular services for piped water) and high human densities provided ideal habitats for *Ae*. *aegypti* and thus the area supported a relatively high and stable year-round population that is atypical of fluctuating seasonal populations that are broadly prevalent in the region. This provides a highly challenging environment, terms of mosquito population, in which to evaluate OX513A technology. Baseline monitoring was initiated in July 2010 using ovitraps which revealed the presence of *Ae*. *aegypti* and absence of *Ae*. *albopictus* in the whole of Itaberaba.

**Fig 1:**
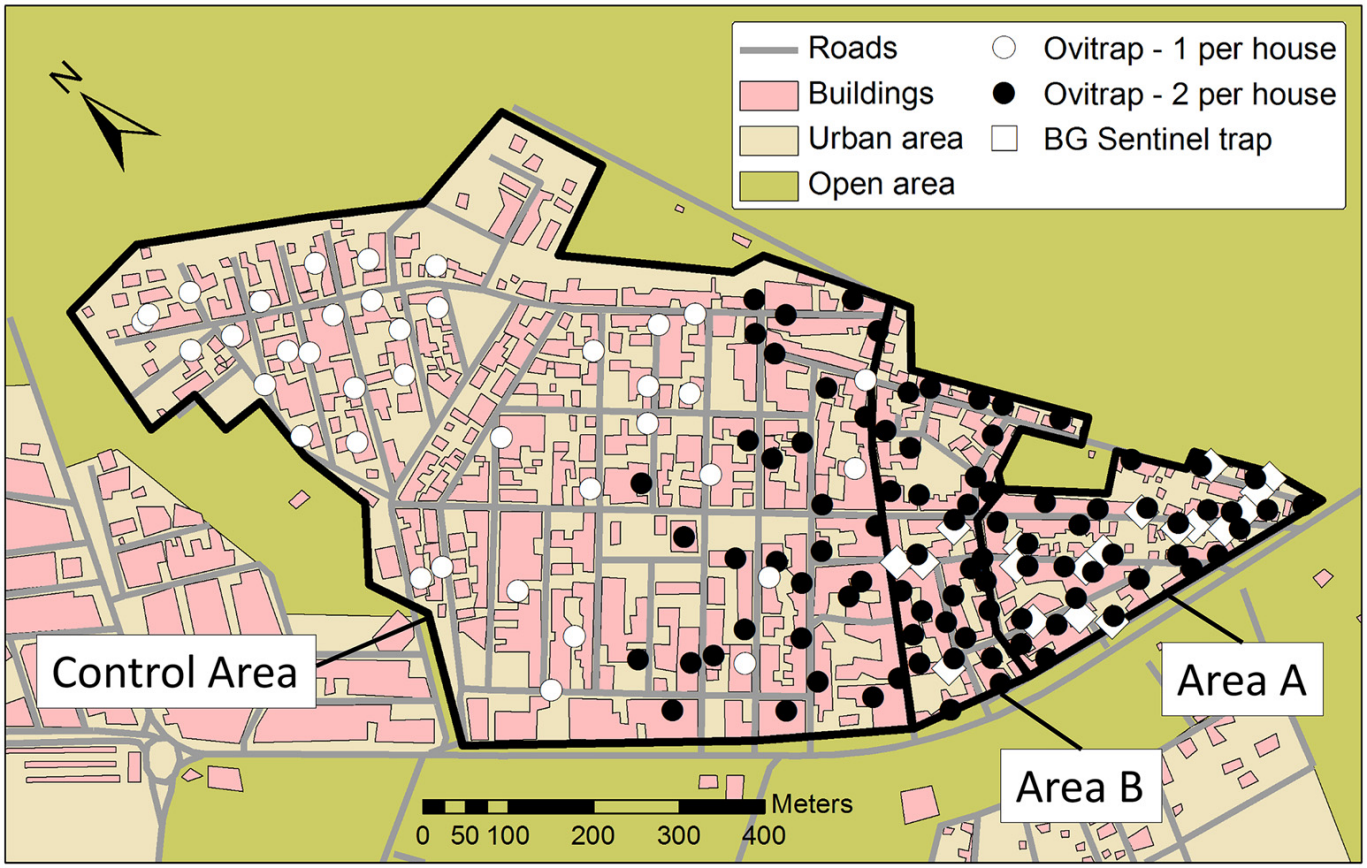
Itaberaba study area. Untreated control area and treatment areas (A and B). Ovitrap distribution is shown for the period 21/11/2011–19/9/2012; open circles = 1 trap house^-1^, solid circles = 2 traps house^-1^. Adult BG Sentinel trap distribution for the period 10/7-25/9/2012 is also shown (open diamonds). Control area = 43.0 Ha, Area A = 5.5 ha, Area B = 5.5 ha.

Human population density was comparable in treated areas A and B with an estimated 165 people ha^-1^ (total 424 houses with 1810 population). Control area density was slightly lower with an estimated 143 people ha^-1^ (1341 houses with 5726 population) (Fig 1). Juazeiro has a semi-arid climate with average annual precipitation of 536 mm falling mostly in warmer summer months (November-April). Throughout the study, conventional local mosquito control was deployed as normal and public heath agents followed standard procedures. Teams of public health agents typically visited homes between 4 and 6 times per year, where they destroyed some breeding sites and treated others with the organophosphate larvicide, temephos. The same team of public health agents were responsible for the whole of Itaberaba suburb, ensuring that underlying conventional mosquito control was similar between the treated and untreated areas of this study.

### Community engagement/regulatory activities

Prior to establishment of the transgenic OX513A line in the mass rearing facility and subsequent open releases, regulatory approvals were obtained from the appropriate Brazilian national regulatory body, Brazilian National Biosafety Technical Commission (CTNBio), for the import permit from the UK to University of São Paulo (Diário Oficial da União (DOU): Extrato de Parecer (EP) 2.031/2009) and for the containment facility for rearing the strain at Biofábrica Moscamed Brasil (DOU: EP 2.577/2010). Approval for releasing OX513A males in the environment was granted in 2010 by CTNBio for five sites, including Itaberaba, around Juazeiro, Bahia (DOU: EP 2.765/2010).

From its inception the project sought to adopt full transparency with a vigorous and proactive community engagement campaign. In addition to national (CTNBio) regulatory approval, consent and support came from regional (Bahia health secretary) and local community leaders (Town Mayor, health secretary and vector control authorities). Implementation included communication via local media (radio, TV and press), community meetings, printed information (posters and leaflets), school presentations, carnival parades, use of small vans with loudspeakers and social media (websites and blogs). Dedicated door-to-door campaigns and ongoing contact with field technicians working in the community provided face-to-face interaction on an individual basis, allowing specific questions to be addressed and for direct feedback and concerns to be aired. Full description of the community engagement will be reported elsewhere.

### Mosquitoes

Transgenic *Ae*. *aegypti* with the OX513A insertion were used during this study [20]. The sensitivity of the OX513A strain to chemical insecticide has been evaluated independent (LSTMH, following WHO protocols). The strain was found to be susceptible to discriminating doses of the insecticide representing all classes commonly used (temephos, permethrin, deltamethrin and malathion,) with the exception of bendiocarb which was found to be non-discriminating for *Ae*. *aegypti*, as the observed levels of resistance were comparable to levels in the New Orleans susceptible reference strain [23] used as a control in the study. The OX513A strain was the same strain previously used for field evaluation in the Cayman Islands [10, 22]. The breeding line was originally imported by Oxitec Ltd. to the University of São Paulo where it underwent laboratory evaluations against Brazilian *Ae*. *aegypti* lines before being transferred to Biofábrica Moscamed Brasil (www.moscamed.com), Juazeiro City.

### Mass rearing of OX513A

Subsequent to obtaining regulatory approval for field release, production of male mosquitoes was conducted at the Moscamed facility in an 84m^2^ laboratory specifically adapted and approved for the purpose. Mass rearing insectaries were maintained at 27°C (+/- 2), 70–90% relative humidity and a 12 hr day/night cycle. A colony of homozygous OX513A was established producing eggs to supply male mosquito production for release. Mosquitoes destined for release were reared to pupae where they were mechanically sorted to remove females [24, 25]. For quality control a minimum of 1500 male pupae from every release batch were individually checked using a microscope to ensure < 1% female contamination. Residual female presence was 0.02% (95% bootstrap CI: 0.016%-0.031%), equivalent to 1 female for every 4,300 males. Weekly quality control checks were made of the transgenic phenotype i.e. expression of the fluorescent marker and lethality in the absence of tetracycline. Detailed methods for production of male pupae followed [26].

### Eclosion and release

**Fig 2:**
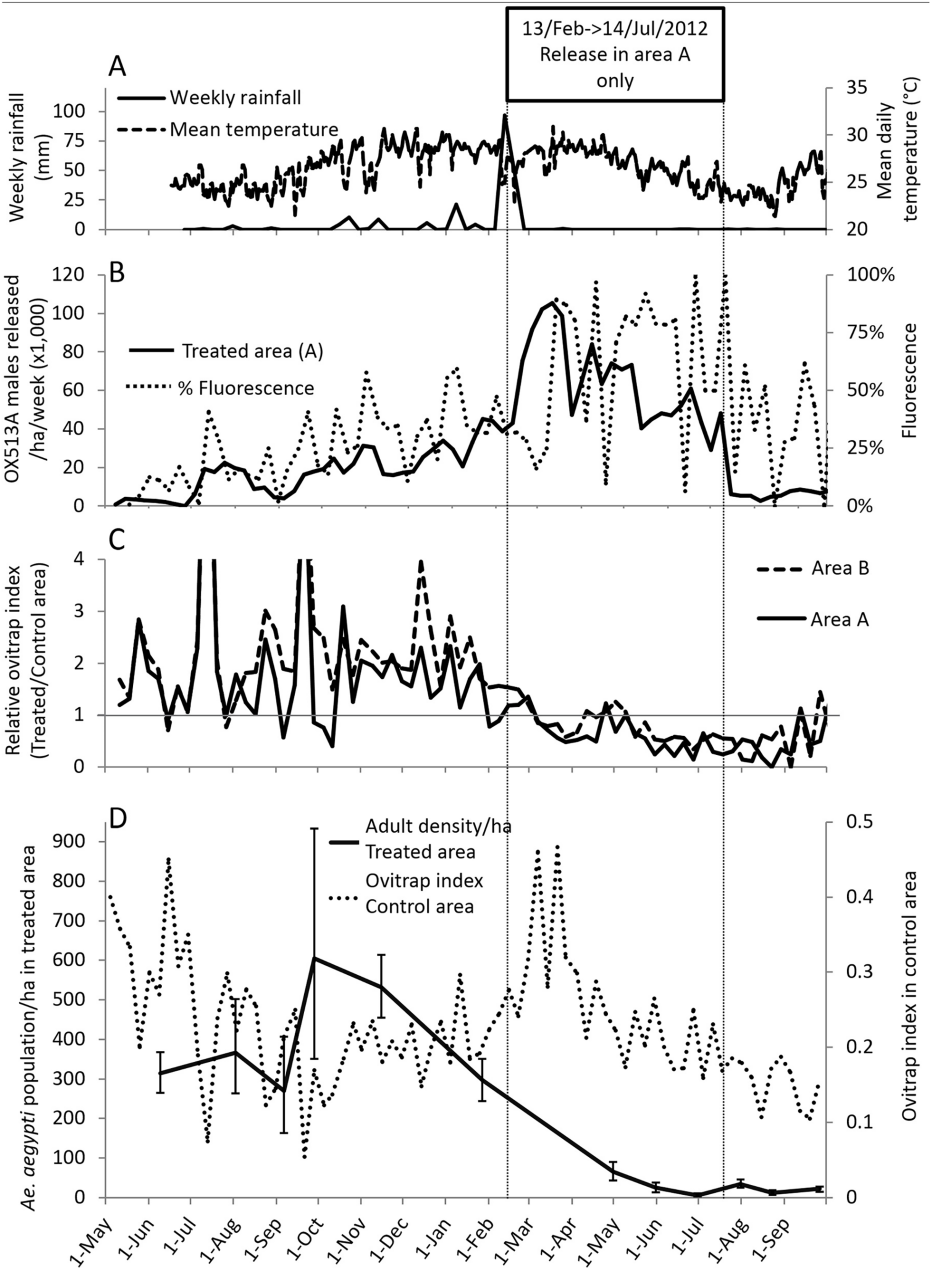
Field data. (A) Weather Data. Temperature and weekly rainfall. (B) Releases and fluorescence. Weekly numbers of adult males released per hectare and percentage of larvae recovered from ovitraps in treated areas with the OX513A transgene as detected by fluorescence. (C) Relative ovitrap index. Relative Ovitrap Index (Treated/Control) in treated areas A and B. The horizontal line represents an equal value of ovitrap index in treated areas and control area. (D) Adult density in treated area and Ovitrap index in control area. Estimated adult wild population of *Ae*. *aegypti* per hectares (error bars = 95% CI) in treated area and ovitrap index in control area. Overall. There is a clear decrease in relative ovitrap index and estimated wild male population from March 2012 to September 2012 following the increase in fluorescence induced by the increased releases while the ovitrap index in the control area remains relatively stable.

Male pupae were aliquoted into release devices (RD) where they eclosed to adults over 24–48 hr before release (for details, see S1 Text). Mosquitoes were dispersed in field site by opening RDs at the rear of a vehicle moving slowly throughout the release area. Releases occurred three times per week. In the initial phase, which we call “rangefinder”, we maintained a constant release rate of males (~ 10,000 per release) for six weeks (total of 185,000 males). This rate was governed largely by production capacity at the time, with priority to releasing a constant rate, rather than sufficient to achieve suppression. The constant release rate provided a relatively stable standing crop of OX513A male population of a known size in the study area against which wild population estimate could be estimated from ratio of OX513A to wild males collected by direct adult trapping, using mark release recapture statistics [27]. Furthermore, the proportion of eggs recovered form ovitraps that were fathered by OX513A males provided an estimate of mating fraction of wild females with OX513A and wild males. This, together with the ratio of OX513A to local males, enabled an estimation of mating competitiveness [10, 28]. Assuming wild populations remained constant, likely release numbers required to achieve a predetermined ratio of released to wild population males in order to achieve suppression could then be estimated from the release rates and corresponding mating competitiveness estimate observed in the rangefinder phase. Thereafter rearing and distribution systems were optimised and sterile male production capacity increased [26]. Release rates increased in line with production allowing extensive estimation of mating competitiveness and eventually suppression. Following suppression, releases were maintained at a lower level (ca. 10 times lower) designed to counter resurgence of population (Fig 2). In addition, low density releases were initiated in a buffer zone extending 2 city blocks (~150m) from Westerly boundary of area B into previously untreated control area to further mitigate resurgence from migration of wild *Ae aegypti* from adjacent populated untreated area. For details see S1 Text, S1 and S2 Figs, and S1 Table.

### Monitoring

Ovitraps were checked and replaced weekly. Ovitrap index was calculated as number of egg-positive traps/total number of traps recovered. Additionally, number of eggs collected from each trap were counted allowing average eggs/trap number to be calculated. Direct monitoring of the adult population was conducted initially by aspiration surveys and later with BG-Sentinel traps (Biogents, Regensburg, Germany). For details see S1 Text.

### Statistics

Statistical analyses were performed using R freeware (R Core Team, Vienna, Austria). Population size was estimated using Petersen-Lincoln test [27] as an estimate of the standing crop of released males. The number of pupae per person was determined from the wild mosquito population estimates following the calculations described by Focks et al. [29]. Confidence intervals (95%) for survival and population estimates were calculated by bootstrap (10,000 repeats).

### Ethics statement

All experiments with animal blood were carried out in accordance with the guidelines of the Ethical Principles for Experiment on Animals adopted by Sociedade Brasileira de Ciência de Animais de laboratório (SBCAL) and approved by the Institutional Ethics Review Committee (Comissão de Ética no Uso de Animais—CEUA)-Universidade de São Paulo, protocol #022/11.

The community engagement protocol was approved by the Institutional Review Board in Human Research (Comissão de Ética em Pesquisa com Seres Humanos do Instituto de Ciências Biomédicas/USP) and Comissão Nacional de Ética em Pesquisa—CONEP, protocol #1115.

## Results

### Range finder

The rangefinder was conducted between May 19th and June 29th 2011, a constant rate of 2,800 OX513A males ha^-1^ week^-1^ were released during that period in the treated area (Fig 1, Areas A and B). The sex ratios (male/female) from direct adult sampling were 2.14 and 0.45 in the treated and control areas, respectively. Thus, we estimated a ratio of 3.7:1 (95% bootstrap CI: 3.19–4.41) OX513A males per wild male was achieved, based on deviation in sex ratio from the 0.45 males per female found in the untreated control area.

**Fig 3:**
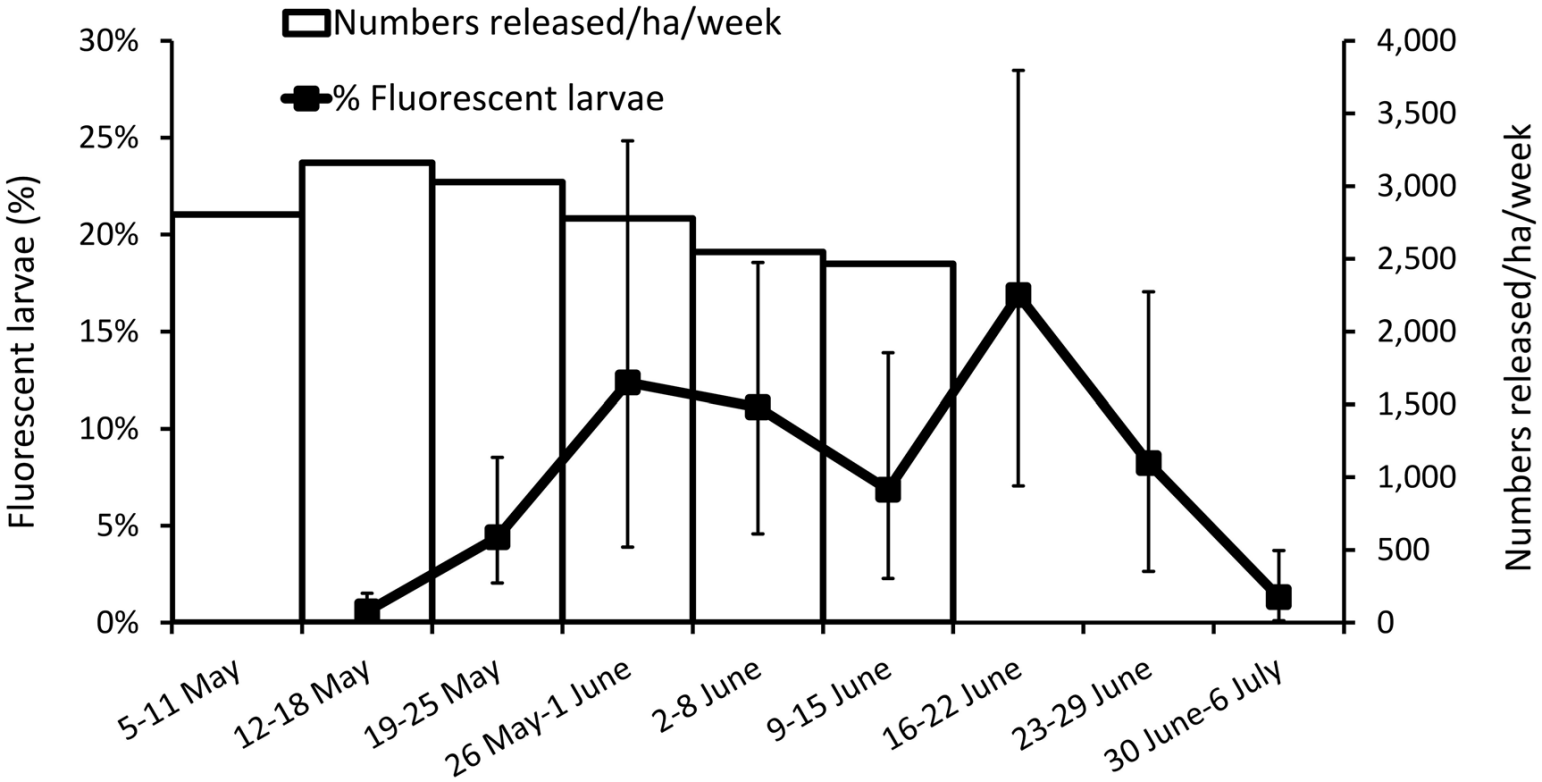
Rangefinder study. Number of OX513A males released and corresponding % fluorescent larvae recovered from ovitraps. Error bars = 95% CI. The releases were fairly constant over the 6-week period, resulting in a presumed stable population of sterile males in the area, after an equilibration period related to adult male lifespan. The first fluorescent larvae were detected one week after the first release and the proportion of fluorescent larvae stabilized around 10% during the third week of releases until two weeks after the releases stopped.

By hatching the eggs from ovitraps and scoring the resulting larvae for fluorescence, we could identify which had an OX513A father—and had therefore inherited a copy of the OX513A transgene, which carries a fluorescent marker—and which had not (Fig 3). By comparing the ratio of OX513A:wild males with the fluorescent:non-fluorescent ratio in hatched eggs (‘fluorescence ratio’) we were able to estimate the mating competitiveness of the released OX513A males. We define field mating competitiveness (C) as the relationship between the numerical density of wild-type (*W*) and OX513A (*O*) males and the relative mating success, such that *C = PW/*(*O*(1-*P*)) where *P* is proportion of sterile mating (= proportion fluorescent larvae) [10, 28]. As expected [22], following releases there was a lag period before fluorescence was detected and then an increase before stabilising 3–4 weeks later. We interpret the lag period as representing the time during which the OX513A male population has accumulated to a steady state (new introductions matched by deaths), and similarly for females emerging late enough to have been exposed to these OX513A males as virgins. However this situation is itself transient as death of OX513A heterozygous offspring will in time start to have an impact on the number of wild mosquitoes, depending on the numbers of OX513A males released and their mating competitiveness in relation to wild males. Females additionally have to blood feed and find oviposition sites. We therefore expected a lag phase comprising a time delay of approximately one week between release of OX513A males and the appearance in ovitraps of eggs that they had fathered and a further delay as the population of females mated prior to this point declined to negligible levels. For the same reason, such eggs were expected for a short period after releases ceased. The data are consistent with these hypotheses (Fig 2). Accounting for a time lag before equilibration of released males and emerging females, we assessed for a 6-week period (19 May-29 June 2011). In total we screened 9,252 larvae of which 943 (10.2% (95% exact binomial CI: 9.6%-10.8%)) were fluorescent. Taking this value together with the male release ratio (3.7:1) implies that the released males had a relative competitiveness of 0.031 (95% bootstrap CI: 0.025–0.036). This is an underestimate due to the potential emigration of OX513A-mated females and immigration of pre-mated wild females.

### Optimising and scaling up production of OX513A

We next developed rearing and distribution systems in Brazil, building on Oxitec’s prior experience from field release in other countries [10, 22, 30]. This involved significant modification and optimisation of methods to make them appropriate for the Brazilian environment, including local sourcing of material [26]. Adult male production correspondingly increased from approximately 30,000 per week during the rangefinder to 540,000 per week in early 2012 [26].

### Estimation of release numbers required for suppression

In addition to refining rearing methods and associated assessment of the field performance of the released mosquitoes, population suppression was a key endpoint of the release program as this would validate the technology in a Brazilian setting. Sterile-male methods such as the OX513A male release control strategy will successfully suppress the target population if a sufficient proportion of females mate sterile males. This threshold mating fraction required for local elimination in the absence of immigration can be estimated from models of mosquito population dynamics [31] as 13–57% [20, 22]. The minimum and maximum threshold mating fraction (13–57%) are derived from modelling a range of values for key parameters driving population dynamics including density depended survival of larvae and reproductive rate. Field experience in Grand Cayman suggested that in that location the threshold mating fraction with OX513A males was towards the lower end of this range, as significant suppression was observed at an estimated mating fraction of 12% [10]. While this does not show that the critical mating fraction was less than 12%, as release rates somewhat below the threshold level may still give significant suppression, it does suggest that the threshold is not at the upper end of the model estimates. We therefore aimed to achieve a mating fraction of 50%, reasoning that even if the population dynamics were somewhat different than in Grand Cayman this would likely be sufficient to achieve suppression. From the rangefinder study we predicted that we would have needed a nine-fold increase in release rate, from 2,800 to 25,000 ha^-1^ week^-1^, to achieve a target 50% mating fraction, assuming mating competitiveness and wild populations remained the same.

### Suppression phase

Up to 11th February 2012 we released into areas A and B (Fig 1), comprising 11 ha in total. However, despite improvements in rearing over the period, in this highly infested area we were unable to produce enough OX513A males with the available resources to consistently maintain a mating fraction of 50%, as judged by the percentage of fluorescent larvae. We therefore reduced the release area to an area of 5.5 ha (Fig 1A). As expected, the fluorescence ratio increased correspondingly.

Actual release numbers, observed mating fraction (% fluorescent larvae), and ovitrap index—a measure of population density—are presented in Fig 3B–3D. The ovitrap index in the untreated area remained relatively stable, showing little seasonal variation. Evaluation of impact of any treatment should be assessed in relation to untreated control areas thereby controlling for fluctuation resulting from local environmental factors such as rainfall and temperature. Ovitrap indices for treated areas A and B are correspondingly presented as relative ovitrap index (ovitrap index in treated area divided by ovitrap index in control area, Fig 3C). In 2011 the ovitrap indices in the treated areas were substantially higher than in the untreated area, with a relative mean ovitrap index of 1.61 (95% bootstrap CI: 1.34–1.91) and 1.93 (95% bootstrap CI: 1.67–2.24) for A and B respectively. As treatment started to take effect, ovitrap indices declined in the treated areas relative to the untreated area, with relative mean ovitrap indexes of 0.35 (95% bootstrap Cl: 0.26–0.45) and 0.49 (95% bootstrap CI: 0.36–0.61) for area A and B respectively from June 2012 onwards. This represented significant reduction (One-way ANOVA: df = 1, P<0.001) in relative ovitrap indices of 78% (95% bootstrap CI: 70.5%-84.8%) and 75% (95% bootstrap CI: 66.5%-81.7%) for areas A and B, respectively.

Mating competitiveness is a key performance measure for released males; estimates of net field mating competitiveness of the released males ranged between 0.0004–0.047 (S2 Table). Mean competitiveness as estimated by this method declined substantially with suppression of the wild population (first 5 estimates before suppression mean 0.030, 95% bootstrap CI: 0.020–0.040; last 5 estimates mean 0.008, 95% bootstrap CI: 0.002–0.016). These estimates may be influenced by immigration of pre-mated females; assuming this is constant, as the wild population is suppressed the proportion of positive ovitraps resulting from immigrating pre-mated females increases. This would result in a reduced estimate of mating competitiveness. The pre-suppression mean of 0.030 therefore represents the best estimate of the overall mean mating competitiveness for OX513A in this study. This is consistent with the estimate from the rangefinder (0.031; 95% bootstrap CI: 0.025–0.036).

Although the exact turning point is difficult to pinpoint, it is clear that suppression of the target population, relative to the untreated control area, began at the same time or even just before reducing the area treated (13 Feb 2012). This, combined with ongoing immigration of sterile males from the adjacent Area A, presumably explains that suppression was seen also in Area B. The majority (66%) of area B is < 100m from release points used in area A, and therefore falls under the flight rage of males *Ae*. *aegypti* which typically disperse (mean distance travelled) 30–100m as reviewed by Silver *et al*. [32]. Furthermore, cessation of releases in area B was coupled with a total increase in numbers releases of 41%, which were all concentrated in area A. Analysis of mating fraction in area B, before and after releases stopped, confirmed that there was considerable migration in area B, from releases in adjacent area A, as there was a slight increase in mating fraction from 0.264 (95% bootstrap CI: 0.218–0.325) to 0.326 (95% bootstrap CI: 0.221–0.431). See S1 Text and S4 Fig for more details. Sterile males do not immediately reduce the target population; rather they lead to mortality in the next generation. Therefore, there is a delay of approximately one generation between the release of sterile males and any consequent effect on population size. Mean fluorescence ratio over the prior 6-week period (22 December 2011–1 February 2012) was 43% (95% bootstrap CI: 34.8–52.1%) and corresponded with a mean release rate of 28,644 ha^-1^ week^-1^ (95% bootstrap CI: 24,929–32,102). This corresponds well with the target release rates predicted from the rangefinder.

Ovitraps provide only an indirect measure of the adult population density, reduction of which is the key target for vector control purposes. Though we consider that changes in ovitrap metrics provide good indications of changes in adult density within an area, they provide a poor guide to absolute number [29]. To provide an independent measure of the adult population we used mark-release-recapture methods to estimate the standing crop of wild *Ae*. *aegypti* adults in the trial area [33], exploiting the periodic releases of OX513A males. Adult collection was initially based on aspiration surveys; later on BG Sentinel traps, for which calculations were carried out for trap collections spanning consecutive periods of approximately four weeks. For each period, the average OX513A male standing crop was calculated from numbers released and an estimated daily survival probability (DSP) of 0.49, this DSP estimate being derived from a series of 14 mark-release-recapture studies using dye-marked cohorts. The wild population density was then estimated based on the relative recapture rates of OX513A and wild males.

These experiments provide clear evidence of a reduction in the standing crop of adults (Fig 3D). We observed a 95% (95% bootstrap CI: 92.2%-97.5%) reduction in the estimated *Ae*. *aegypti* adult standing crop from an average of 418 ha^-1^ (95% bootstrap CI: 307–532), prior to January 2012, to 20 ha^-1^ (95% bootstrap CI: 10–29) in area A from May 2012 onwards. As discussed above, this 95% reduction represents an independent and likely more accurate, assessment of the impact on absolute adult population than ovitrap index, and is the most pertinent measure in relation to impact of intervention with regard to reducing the risk of disease transmission.

## Discussion

In this study we demonstrate effective control of a wild population of *Ae*. *aegypti* by sustained releases of OX513A male *Ae*. *aegypti*. We diminished *Ae*. *aegypti* population by 95% (95% CI: 92.2%-97.5%) based on adult trap data and 78% (95% CI: 70.5%-84.8%) based on ovitrap indices compared to the adjacent no-release control area. We estimated mating competitiveness of the released males to be 0.030 (95% bootstrap CI: 0.020–0.040).

These results are similar to the 82% (95% bootstrap CI:69.7–90.0%) suppression achieved by release of OX513A males in a similar study in Grand Cayman [10]. In both cases, the degree of suppression attainable was expected to be limited by immigration of wild mosquitoes from adjacent untreated areas. *Ae*. *aegypti* dispersal is relatively short, with most published mean dispersal distances falling between 30–100 m; estimates for OX513A males also fall in this range [30, 32]. Effects of immigration would therefore likely be limited to a relatively small boundary zone in a larger program, or not even that if the whole of an isolated population were treated. Suppression may also be limited by the persistence of viable eggs laid at an earlier period. These may hatch over a period of months after deposition, depending on environmental conditions. The gradual reduction in the target population from April 2012 onwards may relate to gradual depletion of this egg bank. As for spatial migration, this ‘temporal migration’ would be of very limited consequence for a larger, longer operational control program. The 0.030 mating competiveness observed was also consistent with the estimate of 0.059 (95% bootstrap CI: 0.011–0.21) from the Grand Cayman study [10]. This suggests that differences between these two wild populations, or in environmental parameters between the sites, or in experimental procedures such as rearing and distribution, had little effect on the ability of OX513A males to win mates. Mating competitiveness as measured by this approach includes any effect of the transgene on the released males, the effect of artificial rearing, handling and distribution, and the effect of migration both of pre-mated females into the area and of released males and mated females out of the area. Relatively few estimates of mating competitiveness under open-field conditions have been published, despite the long history of sterile-male methods. In large-scale, successful SIT programmes, field competitiveness of sterile males was estimated at 0.1 for New World screwworm (*Cochliomya hominivorax*) [28, 34] and <0.01 for Mediterranean fruit fly (*Ceratitis capitata*) [35, 36]. It is a well-documented feature of SIT programs that laboratory caged study estimates of mating competitiveness are not representative of results in the field, where competitiveness values are much lower [27]. This is has also been true for OX513A as observed mating competitiveness in the field are lower than results from laboratory [22, 37], and larger semi-field mating cage studies [38] that have consistently shown OX513A males to be highly competitive achieving equal mating success to non-transformed males counterparts.

Mean fluorescence ratio corresponding with period when suppression was observes was estimated to be 43% (95% bootstrap CI: 34.8–52.1%). This falls within model predictions of 13–57% (19) as did the Grand Cayman suppression (12%); variations in that threshold may indicate somewhat different population dynamics, as might be expected between two very different areas.

The target release rate sufficient to achieve suppression should be proportional to the initial wild population. This may be assessed in terms of the ratio of release to wild males. Alternatively the mating fraction achieved represented a more direct impact of a given release rate without need to account for potential differences in mating competitiveness. Results from this and previous [10] studies support the use of target release rate sufficient to achieve >0.5 mating fraction in large operational programs. Conventional sterile insect based programs generally achieve greater efficiency by initiating releases in conjunction with reduced mosquito population, either by utilising naturally occurring seasonality or temporary knockdown with alternate control such as insecticide application. In this study site there was consistently high mosquito infestation (Fig 2) and no targeted controls was used other than that routinely deployed by vector control teams. Despite this we were able to demonstrate efficacy in challenging conditions, with scope for substantial improved efficiency areas with lower mosquito populations and by incorporation with an integrated vector management program.

This study comprised exclusively entomological endpoints. What would be the impact of such striking reduction of vector population density on dengue transmission? Focks *et al*. estimated a disease transmission threshold in relation to pupae per person^-1^ (as a proxy for adult mosquito population), ambient temperature and herd immunity [29, 39]. For a mean temperature of 28°C Focks et al. calculated an epidemic transmission threshold of 0.42, 0.61 or 1.27 pupae per person for initial seroprevalence of 0%, 33% and 67%, respectively. The average temperature during peak dengue transmission season (January-July) was 27.7°C at the Juazeiro field site, and we can assume a moderate to high seroprevelance given the historical high level of dengue incidence reported in the site by residents and public health workers. Using calculations and assumptions given in Focks *et al*. (2000), we estimate that average pupae person^-1^ decreased in our treated area from 0.7 pre-treatment to 0.04 post-treatment, which in their model would be sufficient to prevent epidemic transmission under these conditions, or indeed under the most adverse conditions modelled for a naive population with 0% seroprevalence. The long-term goal for vector control should be to suppress below the transmission threshold even given low herd immunity [8]–our data indicate that release of OX513A males is able to achieve this goal.[38]

## Supporting Information

S1 Text Additional methodological detail and results. (DOCX)

S1 Dataset Raw data set. Ovitrap data including results for screening larvae for expression of fluorescence, numbers of OX513A males released, direct adult trapping data including recapture of marked OX513A males and data presented in Figs 2 and 3, S2 and S4 Figs.
(XLSX)

S1 Fig Itaberaba study area. Ovitrap distribution is shown for the period 21/11/2011–19/09/2012; open circles = 1 trap house^-1^, solid circles = 2 traps house^-1^. Adult BG Sentinel trap distribution for the period 10/07-25/09/2012 is also shown (open diamonds). Area A = 5.5 Ha, B = 5.5 Ha, C = 8.5 Ha, D = 34.5.
(TIF)

S2 Fig Releases in areas A, B and C over duration of study. Weekly numbers of adult OX513A males released per hectare.
(TIF)

S3 Fig Itaberaba study area with release path. Area A = Red, Area B = Blue, Area C = Yellow.
(TIF)

S4 Fig Percentage fluorescent larvae recovered from ovitraps in areas A-D (error bars = 95% CI) during Phase 2 and 3 of study. (TIF)

S1 Table Study phases—Mean weekly OX513A release rate per ha for different phases of study. (DOCX)

S2 Table Mating competitiveness and wild population estimation. Details of the calculations of mating competitiveness and wild population of *Ae*. *aegypti* over the release period. ^φ^ Sex ratio was different for aspiration (0.45) and for BG-Sentinel traps (0.69).
(DOCX)

S3 Table Mean relative ovitrap index and eggs per trap in Areas A-C compared to control area D before and after suppression. % Reduction expressed as reduction in relative ovitrap index from before and after suppression. Upper and lower 95% CI values given in parenthesis.
(DOCX)

## References

[1] Bhatt S, Gething PW, Brady OJ, Messina JP, Farlow AW, Moyes CL, et al The global distribution and burden of dengue. Nature. 2013;496:504–7. 10.1038/nature12060 23563266 PMC3651993

[2] WHO-TDR. Dengue: guidelines for diagnosis, treatment, prevention and control. Geneva: WHO; 2009. 23762963

[3] de Souza RP, Rocco IM, Maeda AY, Spenassatto C, Bisordi I, Suzuki A, et al Dengue Virus Type 4 Phylogenetics in Brazil 2011: Looking beyond the Veil. PLoS Negl Trop Dis. 2011;5(12):e1439 10.1371/journal.pntd.0001439 22216365 PMC3246447

[4] Shepard DS, Coudeville L, Halasa YA, Zambrano B, Dayan GH. Economic Impact of Dengue Illness in the Americas. The American Journal of Tropical Medicine and Hygiene. 2011;84(2):200–7. 10.4269/ajtmh.2011.10-0503 21292885 PMC3029168

[5] Luz PM, Grinsztejn B, Galvani AP. Disability adjusted life years lost to dengue in Brazil. Tropical Medicine & International Health. 2009;14(2):237–46. 19171013 10.1111/j.1365-3156.2008.02203.x

[6] Rodriguez-Barraquer I, Cordeiro MT, Braga C, de Souza WV, Marques ET, Cummings DAT. From Re-Emergence to Hyperendemicity: The Natural History of the Dengue Epidemic in Brazil. PLoS Negl Trop Dis. 2011;5(1):e935 10.1371/journal.pntd.0000935 21245922 PMC3014978

[7] Wilder-Smith A, Foo W, Earnest A, Sremulanathan S, Paton NI. Seroepidemiology of dengue in the adult population of Singapore. Tropical Medicine & International Health. 2004;9(2):305–8. 15040570 10.1046/j.1365-3156.2003.01177.x

[8] Ooi E, Goh K, Gubler D. Dengue prevention and 35 years of vector control in Singapore. Emerging Infectious Diseases. 2006;12:887–93. 16707042 10.3201/10.3201/eid1206.051210 PMC3373041

[9] Lee K-S, Lo S, Tan SS-Y, Chua R, Tan L-K, Xu H, et al Dengue virus surveillance in Singapore reveals high viral diversity through multiple introductions and in situ evolution. Infection, Genetics and Evolution. 2012;12(1):77–85. 10.1016/j.meegid.2011.10.012 22036707

[10] Harris AF, McKemey AR, Nimmo D, Curtis Z, Black I, Morgan SA, et al Successful suppression of a field mosquito population by sustained release of engineered male mosquitoes. Nat Biotech. 2012;30(9):828–30. 10.1038/nbt.2350 22965050

[11] Dyck VA, Hendrichs J, Robinson AS. Sterile insect technique: principles and practice in area-wide integrated pest management. Netherlands: Springer; 2005.

[12] Knipling E. Possibilities of insect control or eradication through use of sexually sterile males. J Econ Entomol. 1955;48:459–62.

[13] Benedict MQ, Robinson AS. The first releases of transgenic mosquitoes: an argument for the sterile insect technique. Trends Parasitol. 2003;19(8):349–55. Epub 2003/08/07. 12901936 10.1016/s1471-4922(03)00144-2

[14] Klassen W, Curtis CF. History of the sterile insect technique In: Dyck VA, Hendrichs J, Robinson AS, editors. Sterile Insect Technique Principles and practice in area-wide integrated pest management. The Netherlands: Springer; 2005 p. 3–36.

[15] Lowe RE, Bailey DL, Dame DA, Savage KE, Kaiser PE. Efficiency of techniques for the mass release of sterile male Anopheles albimanus Wiedemann in El Salvador. American Journal of Tropical Medicine and Hygiene. 1980;29(4):695–703. 7406117 10.4269/ajtmh.1980.29.695

[16] Thomas DD, Donnelly CA, Wood RJ, Alphey LS. Insect population control using a dominant, repressible, lethal genetic system. Science. 2000;287(5462):2474–6. 10741964 10.1126/science.287.5462.2474

[17] Alphey L, Benedict MQ, Bellini R, Clark GG, Dame DA, Service MW, et al Sterile-insect methods for control of mosquito-borne diseases: an analysis. Vector Borne Zoonotic Dis. 2010;10(3):295–311. Epub 2009/09/04. 10.1089/vbz.2009.0014 19725763 PMC2946175

[18] Catteruccia F, Crisanti A, Wimmer E. Transgenic technologies to induce sterility. Malaria Journal. 2009;8(Suppl 2):S7 10.1186/1475-2875-8-S2-S7 19917077 PMC2777329

[19] Atkinson MP, Su Z, Alphey N, Alphey LS, Coleman PG, Wein LM. Analyzing the control of mosquito-borne diseases by a dominant lethal genetic system. Proc Natl Acad Sci U S A. 2007;104(22):9540–5. 17519336 10.1073/pnas.0610685104 PMC1876161

[20] Phuc HK, Andreasen MH, Burton RS, Vass C, Epton MJ, Pape G, et al Late-acting dominant lethal genetic systems and mosquito control. BMC Biol. 2007;5:11 17374148 10.1186/1741-7007-5-11 PMC1865532

[21] Alphey N, Alphey L, Bonsall MB. A Model Framework to Estimate Impact and Cost of Genetics-Based Sterile Insect Methods for Dengue Vector Control. PLoS ONE. 2011;6(10):e25384 10.1371/journal.pone.0025384 21998654 PMC3187769

[22] Harris AF, Nimmo D, McKemey AR, Kelly N, Scaife S, Donnelly CA, et al Field performance of engineered male mosquitoes. Nat Biotech. 2011;29(11):1034–7. 10.1038/nbt.2019 22037376

[23] Aponte HA, Penilla RP, Dzul-Manzanilla F, Che-Mendoza A, López AD, Solis F, et al The pyrethroid resistance status and mechanisms in Aedes aegypti from the Guerrero state, Mexico. Pesticide Biochemistry and Physiology. 2013;107(2):226–34.

[24] Ansari M, Singh K, Brooks G, Malhotra P, Vaidyanathan V. The development of procedures and techniques for mass rearing of Aedes aegypti. Indian J Med Res. 1977;65:(Suppl) 91–9. 615859

[25] Focks DA. An improved separator for separating the developmental stages, sexes and species of mosquitoes. Mosq News. 1980;19:144–7. 10.1093/jmedent/17.6.567 6111610

[26] Carvalho DO, Nimmo D, Naish N, McKemey AR, Gray P, Wilke ABB, et al Mass Production of Genetically Modified Aedes aegypti for Field Releases in Brazil. J Vis Exp 2014;(83):e3579 10.3791/3579 24430003 PMC4063546

[27] Itô Y, Yamamura K. Role of Population and Behavioural Ecology in the Sterile Insect Technique In: Dyck VA, Hendrichs J, Robinson AS, editors. Sterile Insect Technique 2005 p. 177–208.

[28] Mayer DG, Atzeni MG, Stuart MA, Anaman KA, Butler DG. Mating competitiveness of irradiated flies for screwworm fly eradication campaigns. Preventive Veterinary Medicine. 1998;36(1):1–9. 9677623 10.1016/s0167-5877(98)00078-6

[29] Focks D, Brenner R, Hayes J, E D. Transmission thresholds for dengue in terms of Aedes aegypti pupae per person with discussion of their utility in source reduction efforts. Am J Trop Med Hyg 2000;(62):11–8. 10761719

[30] Lacroix R, McKemey AR, Norzahira R, Lim KW, Wong HM, Teoh GN, et al Open Field Release of Genetically Engineered Sterile Male Aedes aegypti in Malaysia. PLoS ONE. 2012;7(8):e42771 10.1371/journal.pone.0042771 22970102 PMC3428326

[31] Dye C. Models for the population dynamics of the yellow fever mosquito, Aedes aegypti. Journal of Animal Ecology. 1984;53:247–68.

[32] Silver JB. Measuring Adult Dispersal In: Silver JB, editor. Mosquito Ecology 2008 p. 1377–424.

[33] Silver JB. Estimating the size of the Adult Population In: Silver JB, editor. Mosquito Ecology 2008 p. 1273–376.

[34] Vreysen MJB. Monitoring sterile and wild insects in area-wide integrated pest management programmes In: Dyck VA, Hendrichs J, Robinson AS, editors. Sterile Insect Technique Principles and practice in area-wide integrated pest management. the Netherlands: Springer; 2005 p. 325–61.

[35] Shelly TE, McInnis DO, Rodd C, Edu J, Pahio E. Sterile Insect Technique and Mediterranean Fruit Fly (Diptera: Tephritidae): Assessing the Utility of Aromatherapy in a Hawaiian Coffee Field. J Econ Entomol. 2007;100(2):273–82. 17461047 10.1603/0022-0493(2007)100[273:sitamf]2.0.co;2

[36] Rendón P, McInnis D, Lance D, Stewart J. Medfly (Diptera:Tephritidae) genetic sexing: large-scale field comparison of males-only and bisexual sterile fly releases in Guatemala. J Econ Entomol. 2004;97(5):1547–53. 15568342 10.1603/0022-0493-97.5.1547

[37] Patil PB, Niranjan Reddy BP, Gorman K, Seshu Reddy KV, Barwale SR, Zehr UB, et al Mating competitiveness and life-table comparisons between transgenic and Indian wild-type Aedes aegypti L. Pest Management Science. 2015:In Press. 10.1002/ps.3873 PMC4657483 25078081

[38] Lee HL, Vasan S, Ahmad NW, Idris I, Hanum N, Selvi S, et al Mating compatibility and competitiveness of transgenic and wild type Aedes aegypti (L.) under contained semi-field conditions Transgenic Research 2012;22:47–57. 10.1007/s11248-012-9625-z 22700207

[39] Focks DA, Alexander N, Villegas E. Multicountry study of Aedes aegypti pupal productivity survey methodology: findings and recommendations Geneva: World Health Organization, 2006 TDR/IRM/DEN/06.1.

