# Supplementary material for: Suppression of a Field Population of Aedes aegypti in Brazil by Sustained Release of Transgenic Male Mosquitoes
Source: PLoS Negl Trop Dis. 2015 Jul 2;9(7):e0003864. doi: 10.1371/journal.pntd.0003864 (PMC4489809; doi:10.1371/journal.pntd.0003864)
Supplement: S1 Text — (DOCX) [file pntd.0003864.s001.docx]

**S1 Text** – Additional methodological detail and results.

**Materials and Methods**

### **Eclosion and Release**

Male pupae were aliquoted into combined pupal eclosion/adult release devices (RD). Each RD consisted of a 1.8 litre (14cm high x 13 cm diameter) clear plastic cylindrical container (Produtos Prafesta®, Brazil) with a large hole in the lid that was covered with fine mesh to allow for air exchange and sugar feeding provided by cotton wool soaked in 10% sucrose solution. For the 16 releases of the ‘rangefinder study’ ~500 pupae were used per RD, thereafter ~1000 pupae were added to each RD. Once adults had eclosed, any remaining water was drained from pots through a slit in the side of the container before release, which took place approximately 48-72 hrs after pupation. Releases occurred 3 times per week. RD’s were transported in large insulated boxes by truck to the release site and opened according to a scheduled release plan. Total numbers of adults released were estimated by subtracting the numbers of dead adults and pupae remaining in RD’s after release, from the initial numbers of pupae added.

### **Monitoring**

A grid of ovitraps spanning treated and untreated control areas was used throughout the study to provide an indirect measure of adult abundance (Fig 1). Numbers of traps increased in accordance with the increasing study scale (S1 Text-Table 1). From 11 May 2011 onward, larvae were scored for the presence of the OX513A gene based on the characteristic red fluorescence phenotype due to the DsRed2 marker [1, 2] (Clontech Laboratories Inc.) using a Leica MZ10F epi-fluorescence microscope (Leica, Wetzlar, Germany). Throughout the release period, non-fluorescent larvae were reared to adults as an additional check for the presence of *Ae. albopictus*; none were detected. Ovitrap index (number of egg-positive traps/total number of traps recovered) based on the number of egg positive traps, rather than larvae identified as *Ae. aegypti*, was used as a representative statistic. This avoids the possibility of inconsistencies in calculated ovitrap index due to variation in egg hatch. Additionally, total number of eggs collected in all traps was recorded allowing mean number of eggs recovered per trap to be assessed as a supplementary measure of *Ae. aegypti* abundance. Ovitraps are designed to mimic natural oviposition sites [3] and consisted of black plastic pots (11.5 x 11 cm) three-quarters filled with clean water with a fibre board paddle (12 x 3cm) protruding above the water line to provide an oviposition substrate. Traps were checked and replaced weekly.

Direct monitoring of the adult population was conducted initially by aspiration surveys and later with BG-Sentinel traps (Biogents, Regensburg, Germany). Aspiration surveys were conducted using locally made hand-held battery powered aspirators (similar to InsectaZooka [www.BioQuip.com](http://www.BioQuip.com)). After obtaining consent from the respective property owner, each building was sampled for a set period of 15 minutes. Aspiration surveys are very labour intensive and intrusive to the local population and were therefore only conducted at periods coinciding with releases of marked cohorts of mosquitoes. For these reasons BG-Sentinels were later introduced as an alternative adult sampling method. From March 2012 BG-Sentinel traps were installed permanently providing continuous adult monitoring in the treated area. Traps were serviced daily in the course of some mark-release-recapture experiments, otherwise weekly. As trap catches are cumulative over time, it is possible to use the mean catch per day statistic for comparative purposes where different service intervals were used.

**Table 1 (S1 Text).** Ovitrap deployment plan in treatment areas A and B and control areas C and D. For subdivision demarcation of control areas refer to **S1 Fig.**

| Trapping  Period |  | Treated Area | | Control Area | |
| --- | --- | --- | --- | --- | --- |
|  |  | A | B | C | D |
| 22/02/2010 - 14/02/2011 | Houses | 7 | 5 | 4 | 5 |
|  | Traps | 7 | 5 | 4 | 5 |
| 21/02/2011-11/05/2011 | Houses | 7 | 5 | 4 | 24 |
|  | Traps | 7 | 5 | 4 | 24 |
| 18/05/2011 - 13/07/2011 | Houses | 30 | 30 | 5 | 35 |
|  | Traps | 60 | 60 | 5 | 35 |
| 20/07/2011 - 14/12/2011 | Houses | 30 | 30 | 23 | 46 |
|  | Traps | 60 | 60 | 23 | 46 |
| 21/12/2011 - 21/03/2012 | Houses | 30 | 30 | 21 | 46 |
|  | Traps | 60 | 60 | 41 | 57 |
| 21/03/2012- 19/09/12 | Houses | 30 | 30 | 23 | 46 |
|  | Traps | 60 | 60 | 43 | 57 |

**Results**

### **Eggs per trap**

Changes in relative numbers of eggs per trap before and after suppression was analysed as described in main text for ovitrap index over the same time periods (Before; from 04/05/2011 to 28/12/2011, After; From 30/05/2012 to 12/09/2012). We observed a 93% (95% bootstrap CI: 87.0%-96.0%) and 87% (95% bootstrap CI: 78.3%-92.0%) reduction in area A and B respectively.

### **Impact of released OX513A migrating into neighbouring buffer areas.**

Treatment effect in terms of proportion of fluorescent larvae recovered from ovitraps (mating fraction) and subsequent suppression of local *Ae. aegypti* population is expected to extend into a ‘buffer’ area adjacent to treated areas that fall within the dispersal distance of released males. The extent (distance and impact) of this effect within a buffer area will be influenced by dispersal distance and number of released males relative to local *Ae. aegypti* population in buffer area. In order to assess the impact of OX513A dispersal from release site into adjacent buffer areas we subdivided the study site into 4 areas illustrated in **S1 Fig** including areas A and B (Fig 1) with additional demarcation of a buffer area C adjacent to area B. Area C comprised 8.5 ha consisting of a 2 city block (130-190 m) strip, running parallel to Westerly boundary of area B. Housing type and density was consistent with areas A and B. These areas received different treatment regimens in four distinct phases over the course of the study (**S2 Fig** and **S1 Table**). Release tracks used for release of OX513A males in different phase of study are illustrated in **S3 Fig**. The majority (66%) of area B fell within 100m of release points used in treatment of area A. Similarly 66% of area C fell within 100m of release points used for treatment of adjacent area B. As *Ae. aegypti* dispersal range is typically 30-100m [4,5] we would expect significant migration of OX513A males into untreated area C from area B in phase 2, and into untreated area B during phase 3 where releases were restricted to area A only.

### **Fluorescent larvae recovered**

The proportion of fluorescent larvae recovered from ovitraps for each area was analysed separately for phase 2 (releases in areas A and B) and Phase 3 (releases in area A only). Dispersal of OX513A gene radiating out from release site, as detected by recovered fluorescent larvae, represents combined dispersal of released OX513A males and subsequent dispersal of mated females.

In Phase 2, there is clear evidence of gradient in mating fraction with highest levels in area A (32%, 95% bootstrap CI: 25.8%-38.9%) closely followed by B (26%, 95% bootstrap CI: 20.8%-32.5%), with areas C (12%, 95% bootstrap CI: 7.8%-17.4%) and D (3%, 95% bootstrap CI: 0.4%-9.0%) substantially lower than both A and B (**S4 Fig**). Observed higher mating fraction in area A compared to area B may be expected as area A was more insulated from immigration pressure, being relatively isolated on 2 sides. This gradient became more apparent in Phase 3 where released in area B stopped while total release number increased 41% resulting in a 177% increase in release rate in area A (**S1 Table**). Correspondingly, average weekly % fluorescent larvae recovered in area A doubled from 32% to 64% (95% bootstrap CI: 50.4%-76.1%) from Phase 2 to 3. Although there were no releases in area B in phase 3, the % fluorescent larvae also rose marginally from 26% to 33% (95% bootstrap CI: 22.1%-43.1%) compared to Phase 2. This provides supporting evidence that dispersal of OX513A males and OX513A mated wild females from area A into area B was sufficient to sustain a substantial mating fraction leading to observed suppression of population.

### Suppression of wild population in areas A-C

Impact of suppression was assessed separately in each area A-C for both Ovitrap index and average eggs per traps. Impact of treatment was assessed, as described for ovitrap index in the main text, in terms of relative change in relation to a control area, with area D serving as comparator untreated control, such that;

Relative change =$1-\frac{\left( \frac{T_{a}}{U_{a}} \right)}{\left( \frac{T_{b}}{U_{b}} \right)}$

Where:

**T_b_** : Mean *Ae aegypti* Index in the **treated** area **before** treatment impact

**U_b_** : Mean *Ae aegypti* Index in the **untreated** area **before** treatment impact

**T_a_** : Mean *Ae aegypti* Index in the **treated** area **after** treatment impact

**U_a_** : Mean *Ae aegypti* Index in the **untreated** area **after** treatment impact

The time period ‘before’ treatment impact was same as described to assess relative Ovitrap index in manuscript (data collected in 2011). The period ‘after’ consisted of 8 weeks of Phase 3, before resumption of releases in area B, and additional releases started in buffer area C. As expected there was clear evidence of a gradient in suppression effect (**S3 Table**) in line with that observed for % fluorescence (**S4 Fig**).

### **References**

1. Lukyanov KA, Fradkov AF, Gurskaya NG, Matz MV, Labas YA, Savitsky AP, et al. Natural animal coloration can Be determined by a nonfluorescent green fluorescent protein homolog. The Journal of biological chemistry. 2000;275(34):25879-82.

2. Matz MV, Fradkov AF, Labas YA, Savitsky AP, Zaraisky AG, Markelov ML, et al. Fluorescent proteins from nonbioluminescent Anthozoa species. Nat Biotech. 1999;17(10):969-73.

3. Silver JB. Mosquito Ecology - Field Sampling Methods. Third ed2008. 1477 p.

4. Silver JB. Measuring Adult Dispersal. In: Silver JB, editor. Mosquito Ecology2008. p. 1377-424.

5. Lacroix R, McKemey A, Raduan N, Wee L, Alphey L. Open Field Release of Genetically Engineered Sterile Male Aedes aegypti in Malaysia. PlosOne. 2012;7(8).
