# Supplementary figures and images for: Suppression of a Field Population of Aedes aegypti in Brazil by Sustained Release of Transgenic Male Mosquitoes
Source: PLoS Negl Trop Dis. 2015 Jul 2;9(7):e0003864. doi: 10.1371/journal.pntd.0003864 (PMC4489809; doi:10.1371/journal.pntd.0003864)

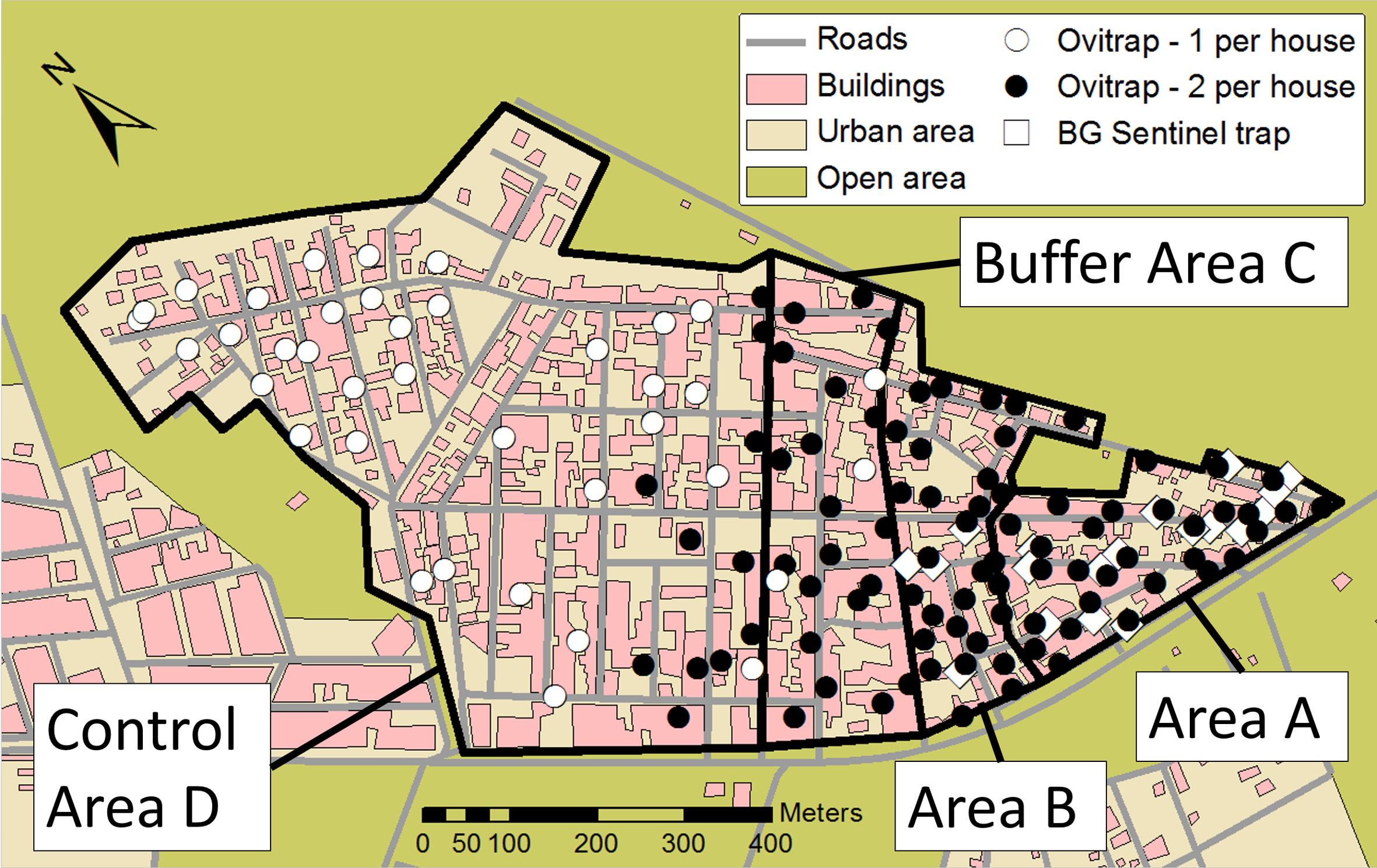

Supplement: S1 Fig — Ovitrap distribution is shown for the period 21/11/2011–19/09/2012; open circles = 1 trap house-1, solid circles = 2 traps house-1. Adult BG Sentinel trap distribution for the period 10/07-25/09/2012 is also shown (open diamonds). Area A = 5.5 Ha, B = 5.5 Ha, C = 8.5 Ha, D = 34.5. (TIF) [file pntd.0003864.s003.tif]

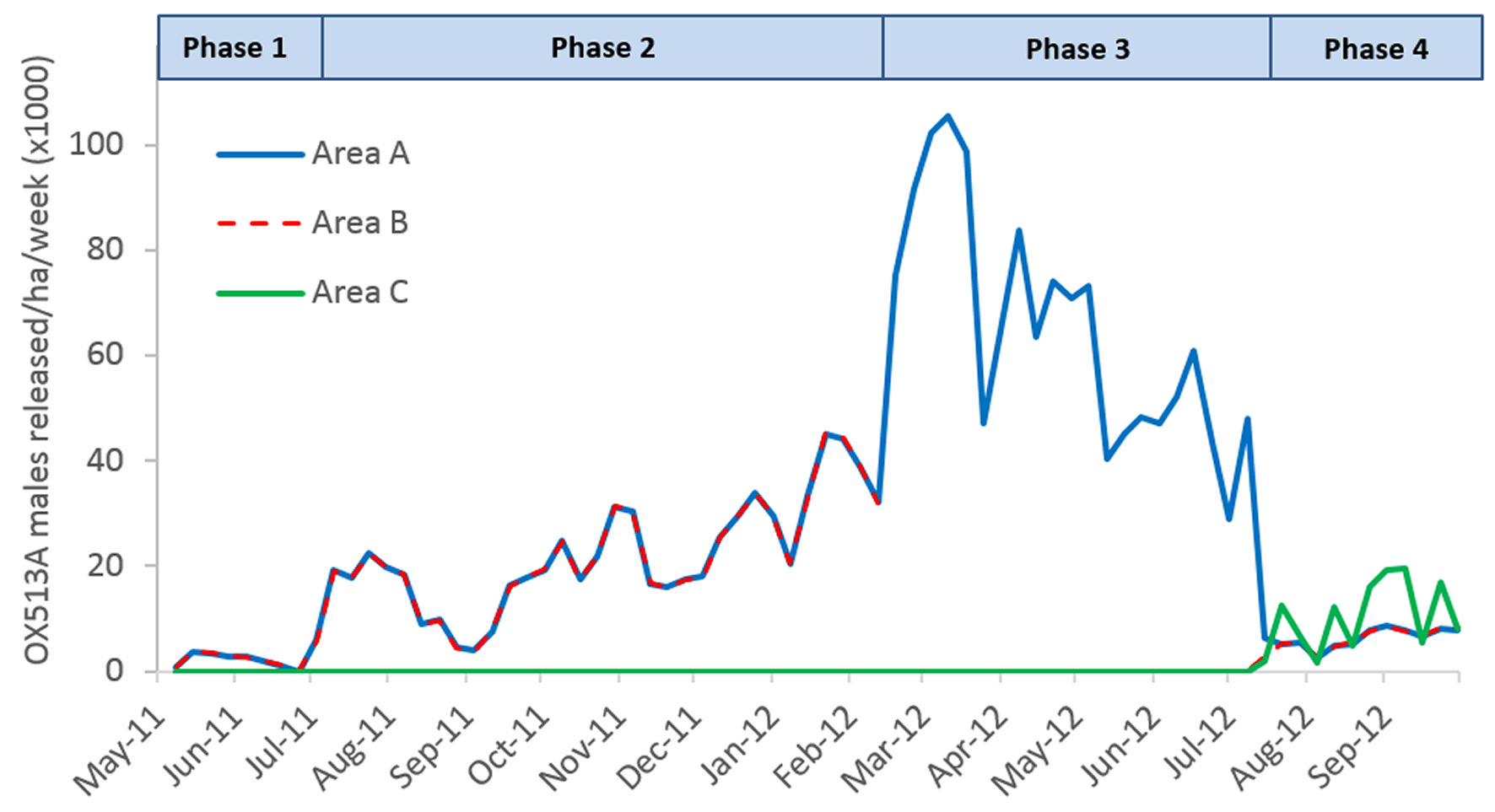

Supplement: S2 Fig — Weekly numbers of adult OX513A males released per hectare. (TIF) [file pntd.0003864.s004.tif]

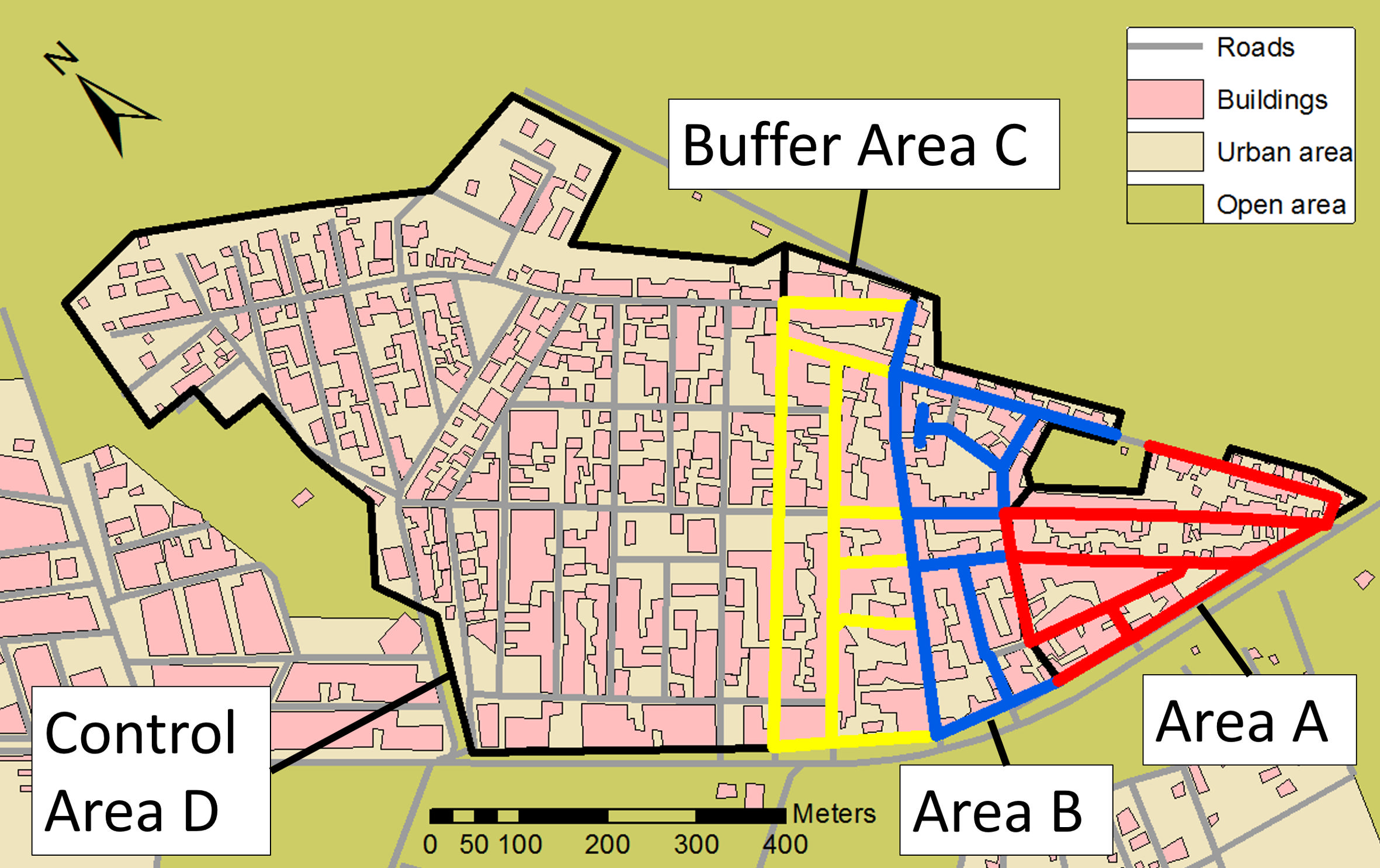

Supplement: S3 Fig — Area A = Red, Area B = Blue, Area C = Yellow. (TIF) [file pntd.0003864.s005.tif]

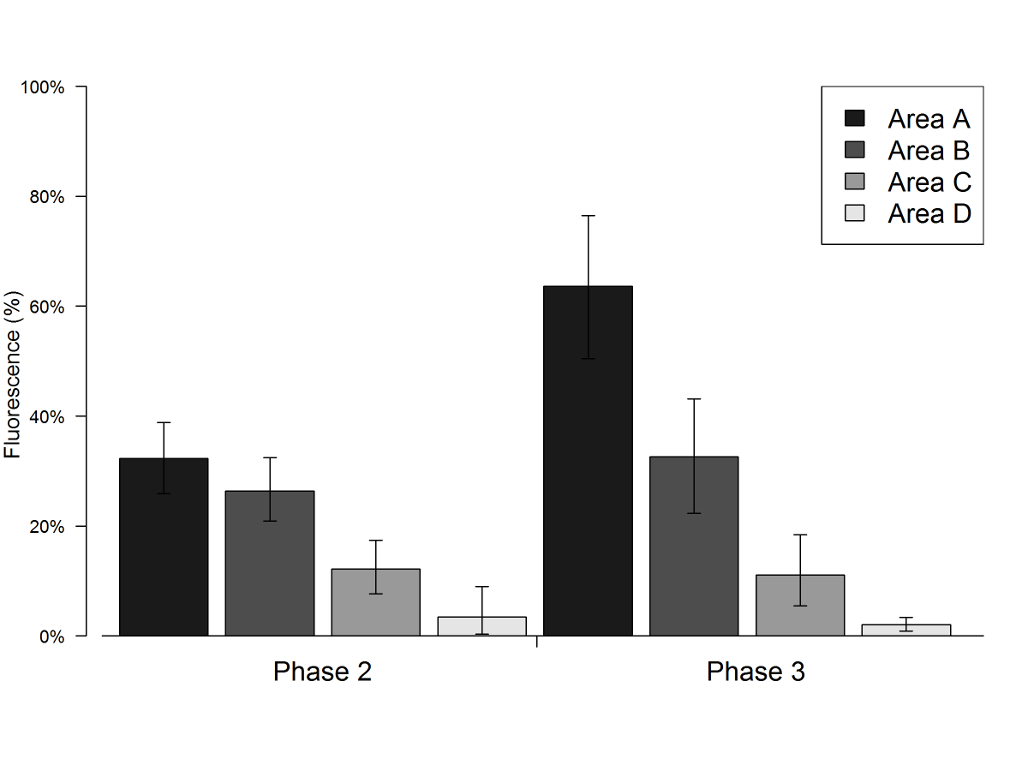

Supplement: S4 Fig — (TIF) [file pntd.0003864.s006.tif]
