# Supplementary material for: Suppression of a Field Population of Aedes aegypti in Brazil by Sustained Release of Transgenic Male Mosquitoes
Source: PLoS Negl Trop Dis. 2015 Jul 2;9(7):e0003864. doi: 10.1371/journal.pntd.0003864 (PMC4489809; doi:10.1371/journal.pntd.0003864)
Supplement: S1 Table — (DOCX) [file pntd.0003864.s007.docx]

| Phases  (Date) | Description | Area A | Area B | Area C |
| --- | --- | --- | --- | --- |
| Phase 1  (7/5/2011 -  13/6/2011) | Rangefinder - Area A and B received equal release rates | 2,800 | 2,800 | 0 |
| Phase 2  (1/7/2011 - 11/2/2012) | Initial Suppression Phase – Area A and B received equal release rates | 23,000 | 23,000 | 0 |
| Phase 3  (11/2/2012-  14/7/2012) | Targeted Suppression Phase – Treatment was suspended in area B, with all releases concentrated in area A. Total number released increased by 41%, resulting in 177% increase in release rate in area A. | 63,600 | 0 | 0 |
| Phase 4  (14/7/2012-30/9/2012) | Maintenance Phase – Having achieved substantial suppression in Areas A and B, release rates were reduced approximately 10 fold in area A relative to average release rates in Phase 3. At same time release were resumed in area B at same rate as used in area A. We sought to maintain relatively constant maintenance released rate in area A and B. To achieve this, target production was set higher and spare capacity from OX513A mass rearing facility was released in Area C, to form a buffer for re-infestation. It was the intention to maintain this for a prolonged period, however, this was curtailed as primary objective of study to demonstrating suppression had been met, and OX513A production was required for another study to be reported elsewhere. | 6,200 | 6,200 | 11,500 |
