# Supplementary material for: Suppression of a Field Population of Aedes aegypti in Brazil by Sustained Release of Transgenic Male Mosquitoes
Source: PLoS Negl Trop Dis. 2015 Jul 2;9(7):e0003864. doi: 10.1371/journal.pntd.0003864 (PMC4489809; doi:10.1371/journal.pntd.0003864)
Supplement: S2 Table — Details of the calculations of mating competitiveness and wild population of Ae. aegypti over the release period. φ Sex ratio was different for aspiration (0.45) and for BG-Sentinel traps (0.69). (DOCX) [file pntd.0003864.s008.docx]

| Date | | **31/05/2011** | **29/07/2011** | **31/08/2011** | **23/09/2011** | **11/11/2011** | **21/01/2012** | **14/04/2012** | **14/05/2012** | **11/06/2012** | **13/07/2012** | **12/08/2012** | **08/09/2012** |
| --- | --- | --- | --- | --- | --- | --- | --- | --- | --- | --- | --- | --- | --- |
| Study Period | | 1 | 2 | 3 | 4 | 5 | 6 | 7 | 8 | 9 | 10 | 11 | 12 |
| Treatment | | Before | Before | Before | Before | Before |  |  | After | After | After | After | After |
| Release area  (ha) | | A +B  11ha | A +B  11ha | A +B  11ha | A +B  11ha | A +B  11ha | A +B  11ha | A  5.5ha | A  5.5ha | A  5.5ha | A  5.5ha | A  5.5ha | A  5.5ha |
| Adult Trapping Method: AS = Aspiration, BG = BG Sentinel | | AS | AS | AS | AS | BG | BG | BG | BG | BG | BG | BG | BG |
| Total male | $M_{T}$ | 893 | 918 | 171 | 258 | 2363 | 6020 | 11368 | 10402 | 11506 | 7653 | 1889 | 5280 |
| Total Female | $F_{T}$ | 417 | 85 | 55 | 37 | 268 | 174 | 42 | 22 | 5 | 66 | 22 | 63 |
| Sex Ratio | $S_{T}$ *=* $M_{T}$*/*$F_{T}$ | 2.14 | 10.80 | 3.11 | 6.97 | 8.82 | 34.60 | 270.67 | 472.82 | 2301.20 | 115.95 | 85.86 | 83.81 |
| Sex Ratio^φ^ (M/F) in untreated area | $S_{C}$ | 0.45 | 0.45 | 0.45 | 0.45 | 0.69 | 0.69 | 0.69 | 0.69 | 0.69 | 0.69 | 0.69 | 0.69 |
| Estimated # wild male | $M_{W}$*=*$F_{T}$***$S_{C}$ | 189 | 39 | 25 | 17 | 185 | 120 | 29 | 15 | 3 | 46 | 15 | 43 |
| Estimated # OX513A | $M_{R}$*=* $M_{T}$*-*$M_{W}$ | 704 | 879 | 146 | 241 | 2178 | 5900 | 11339 | 10387 | 11503 | 7607 | 1874 | 5237 |
| Over-flooding ratio | *O =* $M_{R}$*/*$M_{W}$ | 3.72 | 22.80 | 5.85 | 14.37 | 11.78 | 49.17 | 391.47 | 684.59 | 3335.74 | 167.13 | 123.50 | 120.52 |
| OX513A larvae | $L_{R}$ | 943 | 1533 | 719 | 874 | 2314 | 2611 | 400 | 566 | 76 | 143 | 80 | 123 |
| Wild larvae | $L_{W}$ | 8309 | 5283 | 3354 | 2432 | 4171 | 4169 | 398 | 135 | 60 | 142 | 103 | 45 |
| Proportion OX513A larvae | *P =* $L_{R}$*/(*$L_{R}$*+*$L_{W}$*)* | 0.10 | 0.22 | 0.18 | 0.26 | 0.36 | 0.39 | 0.50 | 0.81 | 0.56 | 0.50 | 0.44 | 0.73 |
| **Mating Competitiveness** | ***C = (P****$\boldsymbol{M}_{\boldsymbol{W}}$***)/((1-P)***$\boldsymbol{M}_{\boldsymbol{R}}$***)*** | **0.031** | **0.013** | **0.037** | **0.025** | **0.047** | **0.013** | **0.003** | **0.006** | **0.0004** | **0.006** | **0.006** | **0.023** |
|  | *Bootstrap 95%CI lower limit* | 0.0254 | 0.0089 | 0.0223 | 0.0138 | 0.0399 | 0.0104 | 0.0016 | 0.0031 | 0.0000 | 0.0039 | 0.0031 | 0.0139 |
|  | *Bootstrap 95%CI upper limit* | 0.0361 | 0.0174 | 0.0546 | 0.0391 | 0.0549 | 0.0152 | 0.0036 | 0.0097 | 0.0008 | 0.0085 | 0.0104 | 0.0352 |
| Mean OX513A male standing crop/ha | $R_{S}$ | 365 | 2611 | 494 | 2714 | 2557 | 5966 | 10421 | 6925 | 6604 | 2360 | 625 | 1081 |
| Male wild standing crop/ha | $M_{W}$ *=* $R_{S}$*/O* | 98 | 114 | 84 | 189 | 217 | 121 | 27 | 10 | 2 | 14 | 5 | 9 |
| Female wild standing crop/ha | $F_{w}$ *=* $M_{W}$*/*$S_{C}$ | 216 | 252 | 186 | 416 | 315 | 176 | 39 | 15 | 3 | 20 | 7 | 13 |
| **Wild adult *Ae aegypti*/ha** | $\boldsymbol{W}_{\boldsymbol{T}}$ ***=*** $\boldsymbol{M}_{\boldsymbol{W}}\boldsymbol{+F}_{\boldsymbol{w}}$ | **315** | **367** | **270** | **605** | **531** | **297** | **65** | **25** | **5** | **35** | **12** | **22** |
|  | *Bootstrap 95%CI lower limit* | 265 | 263 | 163 | 351 | 455 | 244 | 43 | 13 | 0 | 25 | 7 | 15 |
|  | *Bootstrap 5%CI upper limit* | 367 | 502 | 407 | 933 | 614 | 351 | 90 | 38 | 10 | 46 | 19 | 28 |
