# Supplementary material for: Suppression of a Field Population of Aedes aegypti in Brazil by Sustained Release of Transgenic Male Mosquitoes
Source: PLoS Negl Trop Dis. 2015 Jul 2;9(7):e0003864. doi: 10.1371/journal.pntd.0003864 (PMC4489809; doi:10.1371/journal.pntd.0003864)
Supplement: S3 Table — % Reduction expressed as reduction in relative ovitrap index from before and after suppression. Upper and lower 95% CI values given in parenthesis. (DOCX) [file pntd.0003864.s009.docx]

|  | Area A/Area D | | Area B/Area D | | Area C/Area D | |
| --- | --- | --- | --- | --- | --- | --- |
|  | Ovitrap Index | Mean eggs/trap | Ovitrap Index | Mean eggs/trap | Ovitrap Index | Mean eggs/trap |
| Before suppression  04/05/2011-28/12/2011 | 1.80 (1.47-2.23 | 3.41 (2.33-5.04) | 2.16 (1.81-2.63) | 3.71 (2.73-5.10) | 1.30 (1.00-1.66) | 1.49 (1.05-2.13) |
| After suppression  23/05/2012-18/07/2012 | 0.30 (0.23-0.38) | 0.17 (0.09-0.29) | 0.48 (0.39-0.59) | 0.39 (0.26-0.56) | 0.82 (0.65-1.02) | 0.81 (0.51-1.24) |
| **% Reduction** | **84%** (77.5%-88.2%) | **95%** (90.2%-97.5%) | **78%** (70.8%-83.1%) | **90%** (82.8%-93.7%) | **37%** (11.6%-54.9%) | **46%** (5.0%-69.1%) |
